# Neurofibromin Encoded by the *Neurofibromatosis Type 1* (*NF1*) Gene Promotes the Membrane Translocation of SPRED2, Thereby Inhibiting the ERK Pathway in Breast Cancer Cells

**DOI:** 10.3390/ijms262010072

**Published:** 2025-10-16

**Authors:** Nang Thee Su Pwint, Chunning Li, Tong Gao, Yuze Wang, Masayoshi Fujisawa, Toshiaki Ohara, Masakiyo Sakaguchi, Teizo Yoshimura, Akihiro Matsukawa

**Affiliations:** 1 Department of Pathology and Experimental Medicine, Graduate School of Medicine, Dentistry and Pharmaceutical Sciences, Okayama University, Okayama 700-8558, Japan; ng.theesu@gmail.com (N.T.S.P.);; 2 Department of Cell Biology, Graduate School of Medicine, Dentistry and Pharmaceutical Sciences, Okayama University, Okayama 700-8558, Japan; masa-s@md.okayama-u.ac.jp

**Keywords:** breast cancer, SPRED2, neurofibromatosis type 1, neurofibromin, RAS/RAF/ERK

## Abstract

Neurofibromin (NF) inhibits the RAS/RAF/ERK pathway through its interaction with SPRED1 (Sprouty-related EVH1 domain-containing protein 1). Here, we investigated the functional relationship between NF and SPRED2 in breast cancer (BC). Human BC cell lines were transfected to downregulate or overexpress NF and SPRED2 and subsequently subjected to functional assays. Protein and mRNA levels were analyzed by Western blotting and RT-qPCR, respectively. Protein–protein interactions were examined by immunoprecipitation. Database analyses and immunohistochemistry (IHC) of BC tissues were performed to validate the in vitro findings. Downregulating NF or SPRED2 expression in BC cells enhanced cell proliferation, migration and invasion accompanied by RAF/ERK activation, whereas overexpression produced opposite effects. NF formed a protein complex with SPRED2 and facilitated its translocation to the plasma membrane. By IHC, SPRED2 membrane localization was absent in NF-negative luminal A and triple-negative BC (TNBC) but present in a subset of luminal A BC. By database analyses, both *NF1* and *SPRED2* mRNA levels were reduced in BC tissues, and luminal A BC patients with high expression of both *NF1* and *SPRED2* mRNA exhibited improved relapse-free survival. These results suggest a critical role for the NF–SPRED2 axis in BC progression and highlight it as a potential therapeutic target.

## 1. Introduction

Breast cancer (BC) is one of the most prevalent cancers and remains a leading cause of cancer-related mortality among women. According to the 2022 Global Cancer Statistics report, the incidence rate of BC is 27.8%, and the mortality rate is 15% among women worldwide [1]. In the U.S., BC accounts for approximately 30% of all newly diagnosed cancers in women, and 15% of affected patients die from the disease [2]. BC is classified into four molecular subtypes based on the expression of hormone receptors, including estrogen receptor (ER), progesterone receptor (PR) and human epidermal growth factor receptor 2 (HER2). Triple-negative BC (TNBC) is defined by the absence of ER, PR, and HER2 expression, and is characterized by a more aggressive clinical course, poorer prognosis, and higher rates of metastasis and recurrence compared with other BC subtypes [3]. Elucidating the molecular mechanisms underlying the development and progression of BC, particularly TNBC, remains an important and ongoing global challenge.

Germline mutations in the *neurofibromatosis type 1* (*NF1*) gene cause neurofibromatosis type 1 (NF1), the most common autosomal dominant single-gene disorder, affecting approximately 1 in 3000 live births [4]. Individuals with *NF1* mutations are predisposed to developing various malignant tumors, including BC. Notably, carrying a germline *NF1* mutation increases the risk of BC and cancer-related mortality, particularly among women under 50 years of age [5]. Women younger than 40 years with *NF1* mutations are reported to have a 5.5-fold higher risk of developing BC, and their survival rate is approximately half that of the general population [6]. Somatic *NF1* mutations are enriched in advanced and metastatic BC [7]. *NF1* loss is also identified as a genetic driver event in sporadic BC [8,9], and shallow deletions of *NF1* are observed in up to 27% of BC cases [8]. Interestingly, *NF1* mutations are often absent in the primary tumor but emerge during metastatic progression or in advanced BC [7]. In addition to BC, somatic *NF1* mutations have been observed in other cancers, including colorectal, lung, ovarian, bladder, melanoma, glioblastoma, and leukemia [9]. Using two independent large cohorts, an unexpectedly high prevalence (1 in 450–750) of pathogenic *NF1* mutations was identified, which was associated with a significantly increased incidence of multiple malignancies [10]. These findings suggest that *NF1* mutations contribute to cancer risk in the general population.

The *NF1* gene encodes a protein called neurofibromin (NF), a 320 kDa multidomain protein that has been proposed to interact with numerous signaling proteins [11]. The GTPase-activating protein (GAP)-related domain (GRD), located in the middle segment of NF, inactivates RAS by accelerating the hydrolysis of RAS-bound GTP [12]. When *NF1* is mutated, NF loses its ability to promote the conversion of active RAS–GTP to inactive RAS–GDP, leading to hyperactivation of RAS signaling. Consequently, the downstream RAF/ERK signaling cascade becomes persistently activated, promoting cell growth and survival [13]. Sustained ERK activation plays a critical role in the development and progression of BC [14]. Thus, NF functions as an endogenous negative regulator of the RAS/RAF/ERK pathway.

ERK activation is also negatively regulated by SPRED proteins, including SPRED1 and 2 that suppress the RAS/RAF/ERK pathway [15]. Notably, the Eva/VASP homology 1 (EVH1) domain of SPRED1 interacts directly with NF [16], and this interaction is essential for the inhibitory function of SPRED1 on ERK activation. Recently, the structural organization of the NF–SPRED1–K-RAS complex has been elucidated [17], providing new molecular insight into how NF mediates RAS inactivation through its interaction with SPRED proteins.

Previous studies have demonstrated that SPRED2 expression is downregulated in several cancers, including prostate cancer [18], colorectal cancer [19], advanced urothelial carcinoma [20], hepatocellular carcinoma [21], lung adenocarcinoma [22], and BC [23]. It has also been reported that endogenous SPRED2 suppresses cancer cell motility, epithelial–mesenchymal transition, and apoptosis [19,21], while regulating autophagy [24]. These findings collectively indicated that SPRED2 acts as a critical suppressor of cancer development and progression. Structurally, SPRED2 shares a high degree of similarity with SPRED1, containing an EVH1 domain, a c-kit binding domain (KBD), and a Sprouty-related (SPR) domain. Both SPRED1 and SPRED2 effectively inhibit growth factor-induced ERK activation [25]. In the present study, we aimed to elucidate the functions of NF and SPRED2, as well as their interaction, in BC cells, with particular focus on TNBC subtypes.

## 2. Results

### 2.1. Expression of NF1 and SPRED2 in Human BC Cell Lines

**Figure 1:**
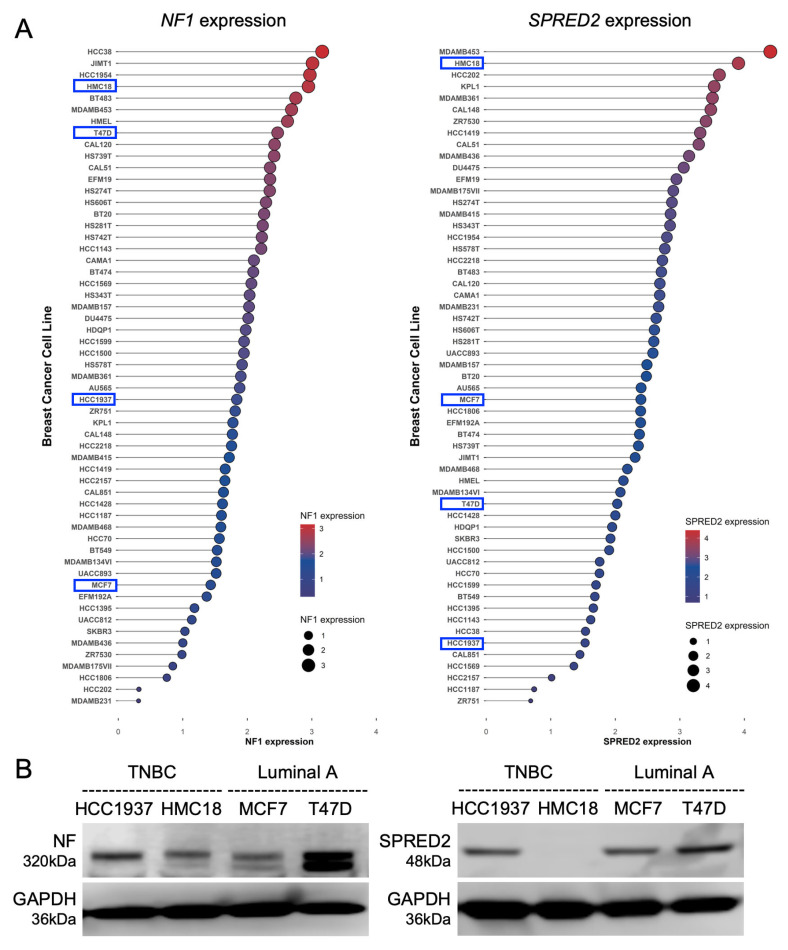
Expression of NF and SPRED2 in human breast cancer cell lines. (**A**) Normalized RNA-seq expression values of *NF1* (**left**) and *SPRED2* (**right**) were obtained from CCLE breast cancer cell lines. Raw read counts were processed in R (v3.36) using the edgeR package (version 3.36) with trimmed mean of M-values normalization and transformed to log_2_ counts per million. Cell lines are ranked in descending order of expression. Dot size and color indicate normalized expression levels. The cell lines selected for subsequent experiments are highlighted by blue boxes. (**B**) Cell lysates were prepared from the indicated cell lines, and NF and SPRED2 protein expression were assessed by Western blotting. Representative images from three independent experiments are shown.

To explore potential involvements of NF and SPRED2 in the phenotypes of BC cells, we first analyzed the expression of *NF1* and *SPRED2* mRNA in 57 human BC cell lines representing different subtypes using raw read count data from the Cancer Cell Line Encyclopedia [26]. Both *NF1* and *SPRED2* mRNA were expressed in most BC cell lines, although the expression levels varied (Figure 1A). We then examined protein expression of NF and SPRED2 by Western blotting in two TNBC cell lines (HCC1937 and HMC18) and two luminal A cell lines (MCF7 and T47D) that were available for this study. NF was detected in all cell lines; however, SPRED2 was absent in HMC18 (Figure 1B), despite its high mRNA expression in the raw read count data (Figure 1A). For subsequent experiments, we selected HCC1937 (TNBC) and MCF7 (luminal A) cells because both proteins were detectable in these cells. The molecular subtypes of MCF7 and HCC1937 cells were further confirmed by immunocytochemistry and Western blotting (Figure S1).

### 2.2. NF and SPRED2 Downregulates BC Cell Proliferation, Migration and Invasiveness

**Figure 2:**
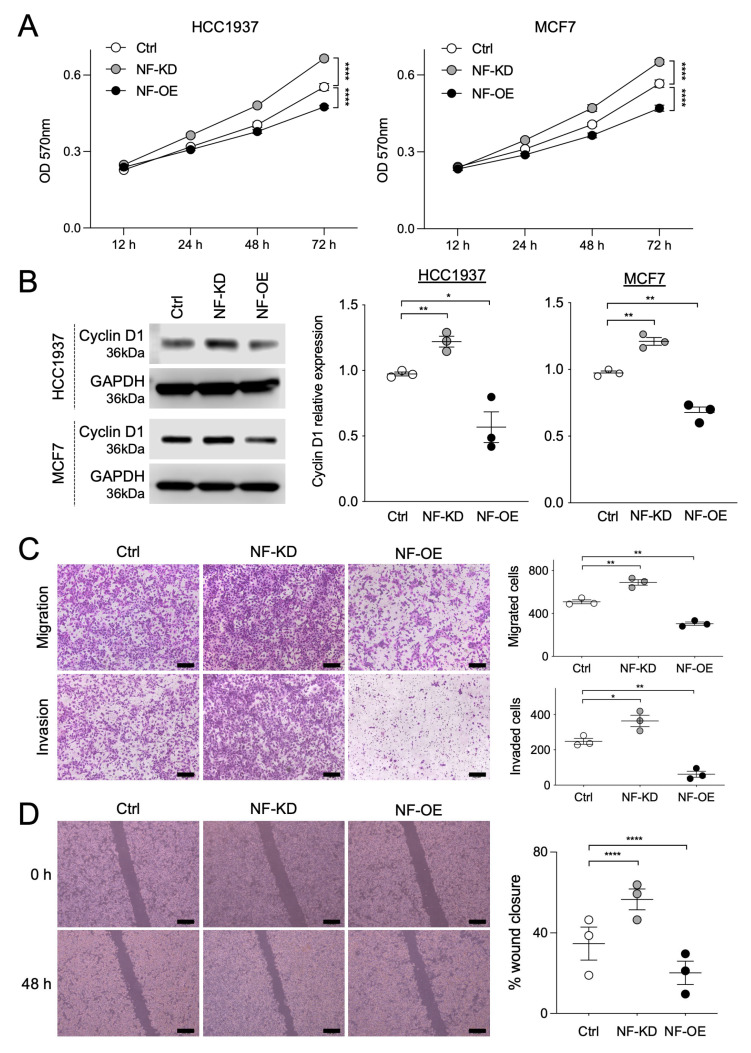
NF negatively regulates BC cell proliferation, migration, and invasiveness. NF was knocked down (NF-KD) or overexpressed (NF-OE) in HCC1937 and MCF7 cells. (**A**) Cell proliferation was evaluated by MTT assay in each cell line. (**B**) Cell lysates were prepared from control (Ctrl), NF-KD and NF-OE cells, and cyclin D1 expression was analyzed by Western blotting. Representative images are shown on the left, and the band intensities were quantified and semi-quantitated from three independent experiments on the right. Cyclin D1 expression levels were normalized to GAPDH. (**C**) Cell migration and invasion assays were performed using HCC1937 cells. Representative images are shown on the left (scale bars: 100 μm). For quantification, cells in three randomly selected low-power fields (20× magnification) per membrane were counted (three independent experiments). (**D**) Scratch assays were performed using MCF7 cells. Representative images captured with an inverted microscope are shown on the left (scale bars: 100 μm). The wound gap distance was measured at the indicated time points using Image J software (v1.54f) (three independent experiments). * *p* < 0.05, ** *p* < 0.01, **** *p* < 0.0001 (two-tailed unpaired *t*-test).

To investigate whether NF negatively regulates BC cell behaviors, we knocked down or overexpressed NF in HCC1937 and MCF7 cells using small interfering RNA (siRNA) or an expression plasmid, respectively. Efficient knockdown or overexpression of NF was confirmed by Western blotting (Figure S2). Compared with the controls, NF knockdown increased the proliferation of both cell lines, whereas NF overexpression decreased the proliferation (Figure 2A). Consistently, the expression of cyclin D1, a proliferation marker, increased upon NF knockdown and decreased with NF overexpression (Figure 2B). Moreover, NF knockdown enhanced the migration and invasion in HCC1937 cells and accelerated wound closure in MCF7 cells, whereas NF overexpression exhibited the opposite effects (Figure 2C,D). These results strongly suggested that NF negatively regulates BC cell proliferation, migration, and invasion.

**Figure 3:**
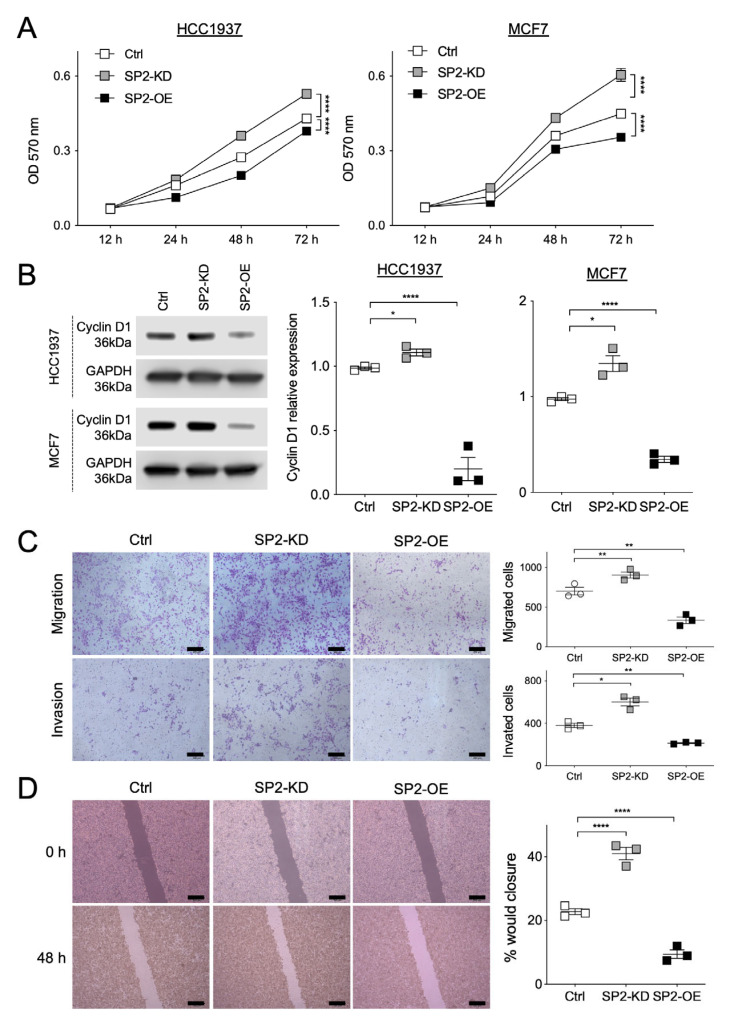
SPRED2 negatively regulates cancer cell proliferation, migration, and invasiveness. SPRED2 was knocked down (SP2-KD) or overexpressed (SP2-OE) in HCC1937 and MCF7 cells. (**A**) Cell proliferation was evaluated by MTT assay in each cell line. (**B**) Cell lysates were prepared from control (Ctrl), SP2-KD and SP2-OE cells, and cyclin D1 expression was analyzed by Western blotting. Representative images are shown on the left, and band intensities were quantified and semi-quantitated from three independent experiments on the right. Cyclin D1 expression levels were normalized to GAPDH. (**C**) Cell migration and invasion assays were performed using HCC1937 cells. Representative images are shown on the left (scale bars: 100 μm). For quantification, cells in three randomly selected low-power fields (20× magnification) per membrane were counted (three independent experiments). (**D**) Scratch assays were performed using MCF7 cells. Representative images captured with an inverted microscope are shown on the left (scale bars: 100 μm). The wound gap distance was measured at the indicated time points using Image J software (three independent experiments). * *p* < 0.05, ** *p* < 0.01, **** *p* < 0.0001 (two-tailed unpaired *t*-test).

We then examined the potential role of SPRED2 in BC cell behavior by knocking down or overexpressing SPRED2 in the same cell lines (Figure S3). Consistent with the effects of NF modulation, SPRED2 knockdown increased BC cell proliferation, migration, invasion, and wound closure, whereas SPRED2 overexpression attenuated these responses (Figure 3). Thus, SPRED2 also acts as a negative regulator of BC cell proliferation, migration, and invasion.

### 2.3. NF and SPRED2 Downregulate BC Cell Proliferation, Migration and Invasiveness via the RAF/ERK Pathway

**Figure 4:**
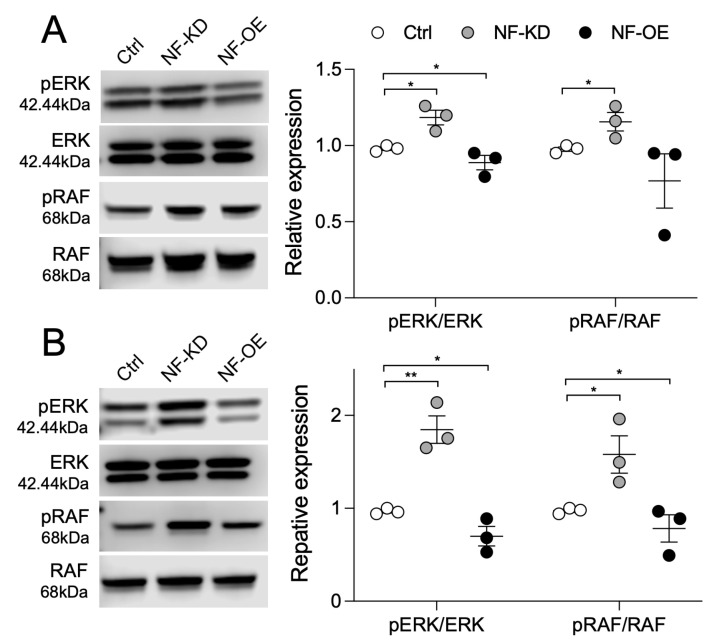
NF negatively regulates ERK and RAF activation. NF was knocked down (NF-KD) or overexpressed (NF-OE) in HCC1937 and MCF7 cells. ERK and RAF phosphorylation in HCC1937 cells (**A**) and MCF7 cells (**B**) were analyzed by Western blotting. Representative images are shown on the left, and band intensities were quantified and semi-quantitated from three independent experiments on the right. * *p* < 0.05, ** *p* < 0.01 (two-tailed unpaired *t*-test).

Previously, we demonstrated that SPRED2 downregulates cancer cell properties by inhibiting the RAS/RAF/ERK pathway [20,21,22]. Since NF is known to inhibit the RAS signaling pathway by promoting the conversion of RAS–GTP to RAS–GDP [13], we examined the phosphorylation status of the downstream signaling molecules, RAF and ERK, in BC cells following NF modulation. As expected, NF knockdown increased the phosphorylation of RAF and ERK, whereas NF overexpression reduced their phosphorylation (Figure 4). Moreover, ERK phosphorylation and cyclin D1 expression in NF or SPRED2 knocked down BC cells were both attenuated by the MEK inhibitor PD98059 (Figure S4). These results indicated that both NF and SPRED2 inhibit BC cell proliferation, migration, and invasion through suppression of the RAF/ERK pathway.

### 2.4. Interaction of NF and SPRED2 in BC Cells

**Figure 5:**
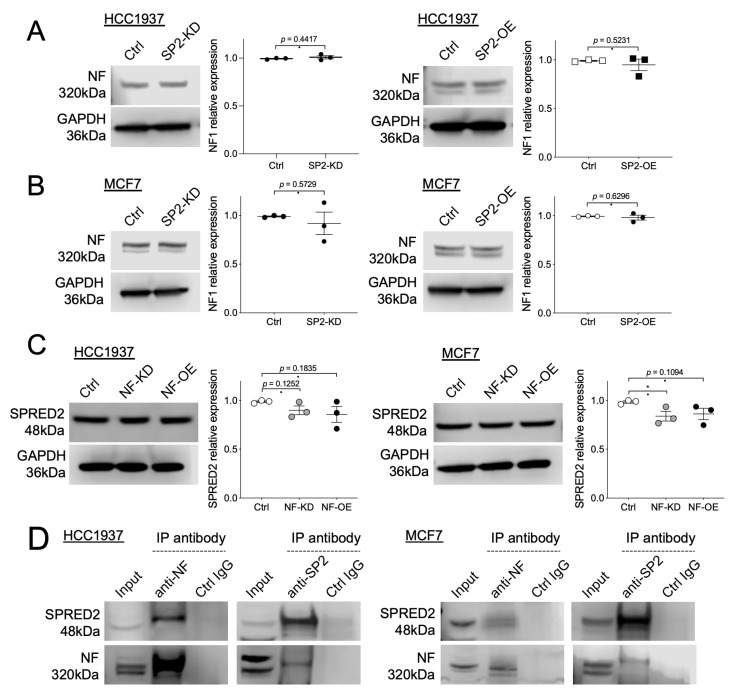
Interaction between NF and SPRED2 in BC cells. (**A**–**C**) NF was knocked down (NF-KD) or overexpressed (NF-OE) in HCC1937 and MCF7 cells. Cell lysates were prepared, and the protein expression of NF and SPRED2 was analyzed by Western blotting. Representative images are shown on the left, and band intensities were quantified and semi-quantitated from three independent experiments on the right. (**D**) Cell lysates (1 mg) from HCC1937 and MCF7 cells were incubated with anti-NF or anti-SPRED2 antibody. The immunoprecipitated proteins were separated by SDS-PAGE and analyzed by Western blotting using anti-SPRED2 or anti-NF antibody, respectively. Representative images are shown. * *p* < 0.05 (two-tailed unpaired *t*-test).

Since NF and SPRED2 exert similar inhibitory effects in BC cells, we asked whether knockdown or overexpression of one protein affects the expression of other protein. SPRED2 modulation did not alter NF levels in both HCC1937 and MCF7 cells (Figure 5A,B). Although knockdown or overexpression of NF resulted in a slight decrease in SPRED2 expression, this reduction was minimal and statistically insignificant in most cases (Figure 5C). These findings indicated that the expression of NF and SPRED2 is largely independent.

As noted above, the interaction between NF and SPRED1 is critical for the inhibition of the RAS/RAF/ERK pathway [17]. Moreover, a recent study demonstrated that NF and SPRED2 can be co-immunoprecipitated from HEK293T cell lysates [27], strongly suggesting that a similar interaction occurs in BC cells to mediate the inhibition of this signaling pathway. To test this possibility, we performed immunoprecipitation using HCC1937 and MCF7 cell lysates and found that NF and SPRED2 were co-immunoprecipitated with either an anti-NF antibody or an anti-SPRED2 antibody (Figure 5D). These results indicated that NF and SPRED2 interact in BC cells and cooperatively regulate the activation of the RAS/RAF/ERK pathway.

### 2.5. NF Promotes the Membrane Translocation of SPRED2

**Figure 6:**
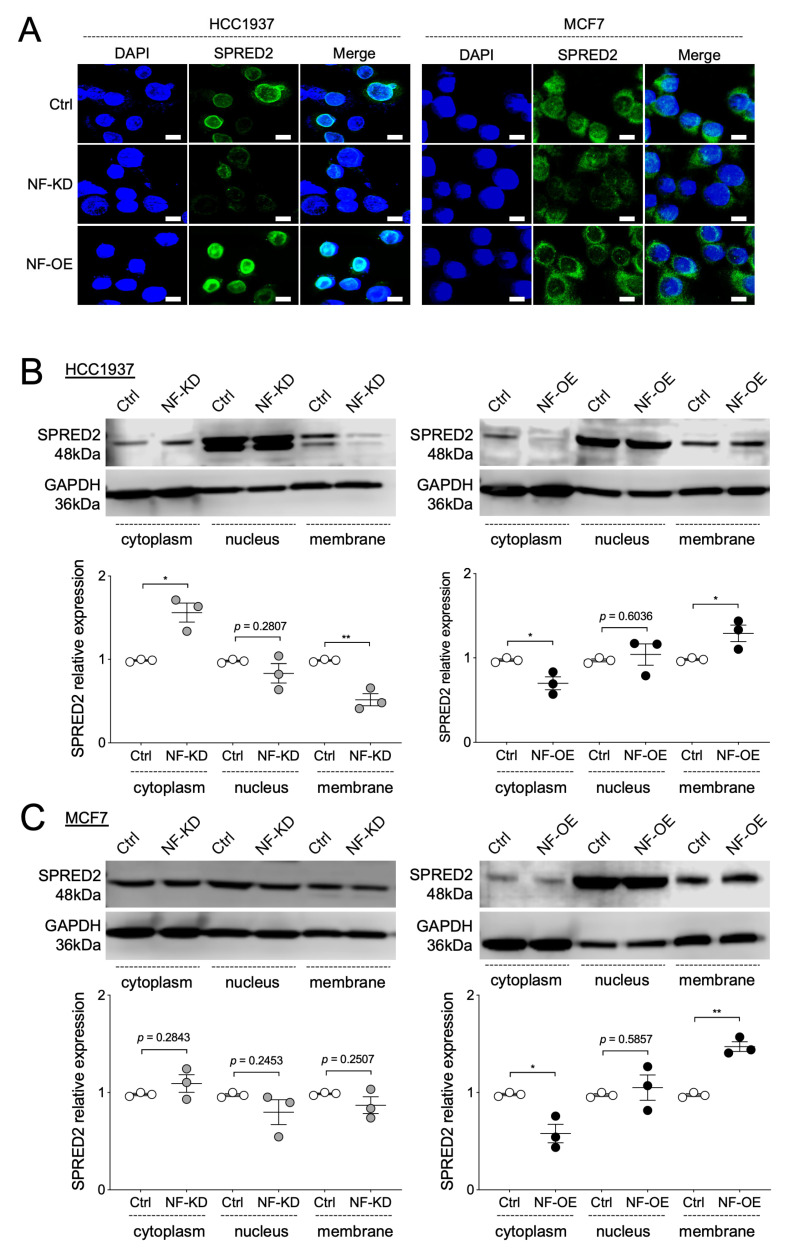
NF promotes the membrane translocation of SPRED2. (**A**–**C**) NF was knocked down (NF-KD) or overexpressed (NF-OE) in HCC1937 and MCF7 cells by transfection with control siRNA (Ctrl) or NF-specific siRNA, and with control plasmid (Ctrl) or NF-overexpression plasmid, respectively. (**A**) SPRED2 localization in HCC1937 (**left**) and MCF7 (**right**) cells were examined by immunofluorescence using a confocal microscope. Representative images are shown (scale bars: 10 μm). (**B**,**C**) Cytoplasmic, nuclear, and membrane protein fractions were isolated from the HCC1937 (**B**) and MCF7 (**C**) cells. The expression of each protein was analyzed by Western blotting. Representative images are shown in the upper panels, and band intensities were quantified and semi-quantitated from three independent experiments in the lower panels. * *p* < 0.05, ** *p* < 0.01 (two-tailed unpaired *t*-test).

SPRED1 recruits NF to the plasma membrane though a two-step process: the N-terminal EVH1 domain of SPRED1 binds NF, while the C-terminal SPR domain anchors SPRED1 to the membrane. This positioning allows NF to access RAS-GTP and promote its conversion to the inactive GDP-bound form, thereby suppressing downstream RAF/ERK signaling. SPRED1 mutations disrupt the recruitment process or impair pathway regulation [28]. SPRED2 also contains the EVH1 and SPR domains [27]. Because SPRED2 and NF form a protein complex in BC cells, we investigated whether NF influences SPRED2 membrane localization. Fluorescence immunolocalization was performed after reducing cytoplasmic proteins as described in Materials and Methods section. NF knockdown reduced membranous SPRED2 staining in both HCC1937 (Figure 6A, left) and MCF7 cells (Figure 6A, right), whereas NF overexpression enhanced membranous staining in these cells (Figure 6A). To further evaluate SPRED2 localization, we analyzed the expression of SPRED2 in cytoplasmic, nuclear, and membrane fractions by Western blotting after NF knockdown or overexpression. In HCC1937 cells, NF knockdown decreased membrane SPRED2, while NF overexpression increased it (Figure 6B, left). By contrast, NF overexpression reduced cytoplasmic SPRED2 and increased membrane SPRED2 (Figure 6B, right). In MCF7 cells, NF knockdown did not significantly affect SPRED2 localization (Figure 6C, left), but NF overexpression decreased cytoplasmic SPRED2 and increased membrane-associated SPRED2 (Figure 6C, right). These results indicate that binding to NF plays a critical role in promoting SPRED2 membrane translocation in BC cells.

### 2.6. NF and SPRED2 Expression in Clinical Specimens

**Figure 7:**
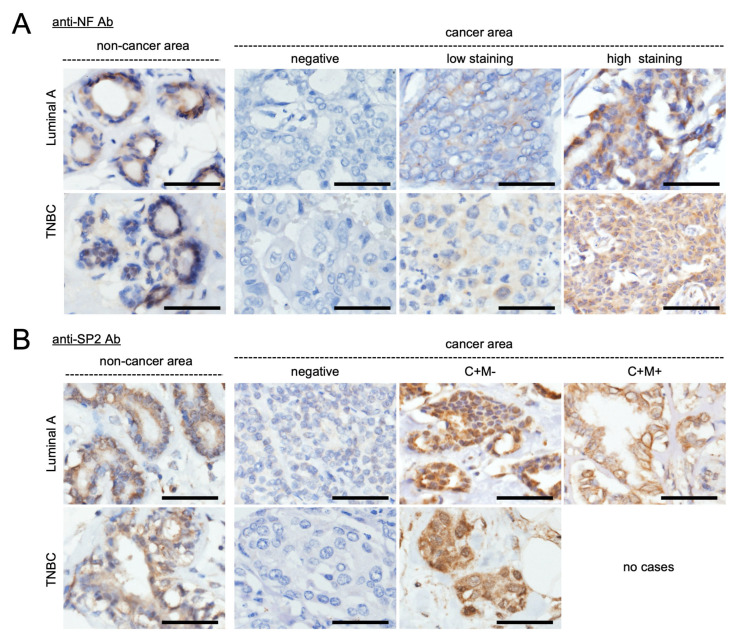
Expression of NF and SPRED2 in BC tissues. A total of 94 invasive BC tissue specimens were stained with anti-NF or anti-SPRED2 antibodies. Representative images of NF (**A**) and SPRED2 (**B**) staining are shown (original magnification, 400×). (**A**) NF expression was classified based on staining intensity as negative, low, or high. (**B**) SPRED2 staining was categorized as negative, cytoplasm positive (C+), membrane negative (M−), or membrane positive (M+). Scale bars: 50 μm.

Next, we investigated the clinical relevance of our in vitro findings by examining NF and SPRED2 protein expression in 94 clinical BC tissue samples (49 TNBC and 45 luminal A cases). Cases were classified as NF-negative if no cytoplasmic staining was observed in the tumor cells. NF staining was validated using internal controls. In non-cancer areas, NF exhibited moderate cytoplasmic staining regardless of TNBC or luminal A status (Figure 7A,B, left). In cancer areas, NF expression was classified into three groups—negative, low, and high—based on staining intensity. Table 1 summarizes the staining patterns for all cases. A significant difference was observed between TNBC and luminal A groups, with high-intensity staining less frequent and negative or low staining more prevalent in TNBC (Table 1). In non-cancer areas of both TNBC and luminal A, SPRED2 was detected in the cytoplasm (Figure 7B, left). In cancer areas, SPRED2 immunohistochemical staining patterns differed significantly between TNBC and luminal A groups (Figure 7B, right; Table 2). Notably, no TNBC cases exhibited membrane-positive SPRED2 staining, whereas 40% of luminal A cases were positive in both the cytoplasm and membrane.

Next, we examined the expression status of NF and SPRED2 staining (Table 3). In NF-negative cases, SPRED2 membrane expression was absent in both TNBC and luminal A cases. Among NF-positive cases, SPRED2 membrane expression was observed in 18 of 35 luminal A cases, but not in any TNBC cases (Table 3). Of the 19 death cases (Table 4), only 3 cases showed SPRED2 membrane expression, whereas 16 cases did not. These findings indicated an association between NF expression and SPRED2 membrane localization in clinical specimens and suggest that SPRED2 membrane expression is important for patient prognosis.

### 2.7. Database Analysis of NF1 and SPRED2 mRNA Expression and Their Prognostic Significance in BC

**Figure 8:**
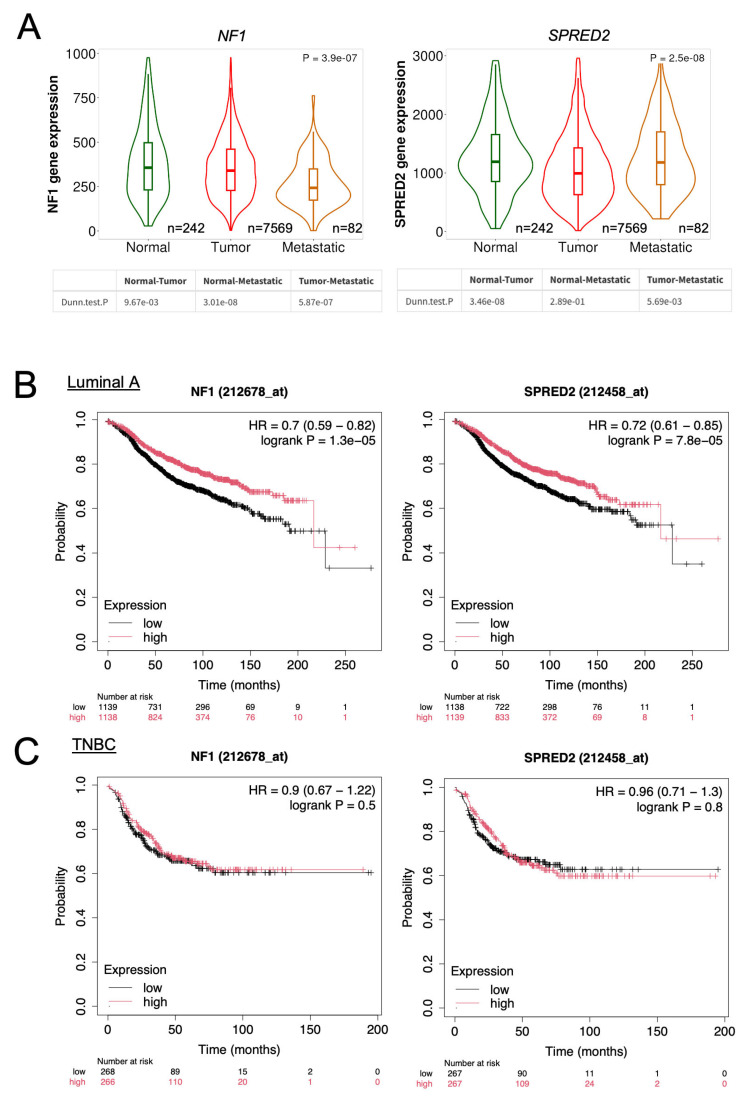
Database analysis of *NF1* and *SPRED2* mRNA expression and their prognostic value in BC. (**A**) *NF1* and *SPRED2* mRNA expression levels in normal breast tissues, primary breast cancer tissues, and metastatic tissues were obtained from TNMplot. Statistical significance was assessed using Dunn’s multiple comparison test. (**B**,**C**) The prognostic significance of *NF1* and *SPRED2* mRNA expression in invasive luminal A BC (**B**) and TNBC (**C**) were evaluated using Kaplan–Meier Plotter. A log-rank *p*-value < 0.05 was considered statistically significant.

Finally, we investigated whether *NF1* and *SPRED2* expression levels could serve as prognostic indicators for BC patients by analyzing public datasets. Compared with normal tissue, *NF1* mRNA expression was significantly reduced in primary BC tumors and further decreased in metastatic cancer (Figure 8A, left). *SPRED2* mRNA expression exhibited a similar pattern (Figure 8A, right). Kaplan–Meier plotter analysis revealed that luminal A BC patients with high *NF1* and *SPRED2* mRNA expression exhibited significantly better relapse-free survival than those with low expression levels (Figure 8B). In contrast, prognosis did not differ between TNBC patients with high versus low NF1 or SPRED2 expression levels (Figure 8C). These findings indicated that high NF1 and SPRED2 expression is associated with a favorable prognosis in luminal A, but not in TNBC patients. This suggests that the relatively low membrane localization of SPRED2 may underlie the lack of prognostic significance observed in TNBC.

## 3. Discussion

Recent studies have underscored the pivotal role of SPRED2 in cancer development and progression, generating considerable research interests. Similarly, NF functions as an endogenous inhibitor of RAS/RAF/ERK pathway and *NF1* mutations are known to have a higher risk of cancerous tumors. In this study, we aimed to investigate the role of NF and its interaction with SPRED2 in BC. Previous studies have primarily examined the independent tumor-suppressive functions of NF1 or SPRED2 in various cancers; however, the cooperative interaction between these proteins and their biological significance in BC remain unclear. Our findings advance the field by demonstrating that NF is required for SPRED2 membrane localization, which is essential for effective inhibition of the RAS/RAF/ERK pathway–a mechanistic link not previously described in BC. Notably, SPRED2 membrane localization was absent in NF-negative tumors and in TNBC cases, but retained in a subset of luminal A BC, suggesting a novel mechanism that may contribute to the aggressive phenotype of TNBC. Collectively, these results represent a conceptual advancement over previous studies focused solely on single-protein functions, emphasizing the critical role of NF–SPRED2 interaction in BC pathology.

A recent study has shown that the loss of *NF1* accelerates breast tumor formation in rats, and that low *NF1* mRNA expression levels in human BC are associated with shorter patient survival times [29]. Furthermore, reduced tumor *NF1* mRNA expression has been linked to poor prognosis in other cancer types, including hepatocellular carcinoma [30], ovarian cancer [31], and colorectal cancer [32]. These findings underscore the importance of elucidating the functions of *NF1* in cancer development and progression. The *NF1* gene comprises 60 exons and produces multiple tissue-specific isoforms through alternative splicing involving additional exons [33]. However, analyzing the *NF1* gene status remains challenging due to its large size, the presence of multiple pseudogenes, and the wide variety of mechanisms that can lead to gene inactivation. Individuals with NF1 typically present with benign cutaneous lesions, such as neurofibromas and café-au-lait macules. Notably, acquired somatic mutations in the *NF1* gene have also been identified in several malignant neoplasms, including BC, that are not associated with neurofibromatosis [9]. The identification of somatic *NF1* mutations in sporadic tumors suggests that the *NF1* gene plays a critical role in tumorigenesis. Using our clinical BC specimens, we observed that approximately one-third of the cases showed negative NF staining. Loss of NF immunoreactivity is known to be associated with biallelic *NF1* gene inactivation [34], suggesting that the NF-negative cases in our study may result from sporadic *NF1* mutations. However, the specific genetic abnormalities underlying these cases in BC remain to be clarified.

Interaction with SPRED1 recruits the tumor suppressor NF to the plasma membrane, thereby enabling NF to inactivate RAS [35]. In the present study, we demonstrated for the first time that NF promotes SPRED2 localization to the membrane. Previous studies have reported that SPRED2 variants lacking the SPR domain fail to translocate to the plasma membrane and downregulate ERK signaling, indicating that the SPR domain is necessary for SPRED2 membrane localization [15,36,37]. This raises the question regarding how NF regulates SPRED2 membrane localization. Under resting conditions, SPRED2 is mainly located in the cytoplasm. Upon growth factor stimulation, SPRED2 translocates to vesicular and membrane compartments, a process that requires its C-terminal SPR domain, as mutants lacking this domain remain in the cytoplasm [38]. It is therefore plausible that certain cytoplasmic factor(s) cooperate with NF to regulate SPRED2 membrane translocation. A recent study demonstrated that RSK2 forms a complex with SPRED2 and directly interacts with its C-terminal SPR domain [27]. Although NF and RSK2 do not appear to interact directly, they may be incorporated into the same complex via cytoplasmic SPRED2. Further in-depth studies are warranted to elucidate this mechanism.

TNBC is known to be more aggressive than the luminal A subtype. In this study, we identified two differences between TNBC and luminal A BC. First, lack of NF expression was observed in 42% of TNBC cases, compared with 22.2% of luminal A cases, indicating that NF expression is lower in TNBC. Second, TNBC cases lacked SPRED2 membrane expression. We previously demonstrated that SPRED2 is localized in the cytoplasm of non-tumor cells and that the presence of SPRED2 on cell membrane suppresses the progression of noninvasive papillary urothelial carcinoma, likely by inhibiting the RAS/RAF/ERK pathway [20]. In the present study, however, membrane-associated SPRED2 expression was not observed in TNBC cases, even in those with positive NF expression. The absence of membrane-associated SPRED2 may therefore contribute to the aggressive phenotype of TNBC. The limitations of our study include the relatively small size of the immunohistochemical dataset and the lack of clinical outcome information for the evaluated patients. Future retrospective or longitudinal research with larger patient cohorts will be essential to validate and further expand upon these results.

In conclusion, we demonstrated here that NF and SPRED2 inhibit BC malignant phenotype by suppressing the RAS/RAF/ERK pathway. NF promotes SPRED2 membrane localization, thereby enabling effective RAF/ERK inhibition. Notably, SPRED2 membrane expression was absent in NF-negative BC, present in a subset of luminal A cases (18/35), and absent in TNBC. Given the aggressive nature and limited treatment options of TNBC, loss of NF–SPRED2 interaction may contribute to its malignant phenotype. Although precision oncology has advanced, conventional chemotherapy remains the mainstay for TNBC, often with suboptimal outcomes [39]. Targeting the RAS/RAF/ERK pathway represents a promising therapeutic strategy [40], and recent studies have investigated bioactive products derived from plants, microbial or marine organisms, as well as nanomaterials, as modulators of this pathway, including in BC [41,42]. Collectively, our findings emphasize the NF–SPRED2 axis as a potential novel therapeutic target, and strategies aimed at restoring or enhancing its function may offer novel approaches for the prevention and treatment of BC, particularly TNBC.

## 4. Materials and Methods

### 4.1. In Silico Analysis

To analyze NF1 and SPRED2 mRNA expression in BC cell lines, raw RNA-seq read count data from the Cancer Cell Line Encyclopedia [26] were processed in R (v3.36) using a custom normalization pipeline. Briefly, the raw read counts were imported into a data frame with gene symbols as row identifiers and cell lines as column identifiers. Normalization was performed using the edgeR package, including library size adjustment with calcNormFactors and transformation to log_2_ counts per million (CPM). Quality control included the removal of genes with low expression (row sums ≤ number of samples). The normalized expression values were visualized using the ggplot2 package. Expression levels of NF1 and SPRED2 across BC cell lines were displayed as lollipop plots, with both point size and color scaled according to expression levels.

Gene chip-based datasets from normal breast tissues, primary breast cancer tissues, and metastatic tissues were obtained from the TNMplot database (https://tnmplot.com/analysis/ (accessed on 1 September 2025)). The prognostic impact of NF1 and SPRED2 mRNA expression in BC was evaluated using data from the Kaplan–Meier Plotter (http://www.kmplot.com (accessed on 16 September 2025)). A log-rank *p*-value < 0.05 was considered statistically significant.

### 4.2. Cell Culture

HCC1937 cells, representing TNBC (JCRB cell bank, Osaka, Japan), were cultured in RPMI 1640 medium (Sigma-Aldrich, St. Louis, MO, USA) supplemented with 10% fetal bovine serum (FBS) (Gibco, Carlsbad, CA, USA), 100 U/mL penicillin, and 100 μg/mL streptomycin, (Sigma-Aldrich). MCF7 cells, representing luminal A BC, were grown in Eagle’s minimal essential medium (low glucose; Sigma-Aldrich) supplemented with 10% FBS, 100 U/mL penicillin, and 100 μg/mL streptomycin. In some experiments, cells were treated with the MEK/ERK inhibitor PD98059 (20 μM; Thermo Fisher Scientific, Waltham, MA, USA) for 24 h. All experiments were performed using mycoplasma-free cells.

### 4.3. Transfection

Transfection was performed in 6-well plates seeded with 2 × 10^5^ cells, using either RNAiMAX (Thermo Fisher Scientific, Waltham, MA, USA) or Lipofectamine 3000 (Life Technologies, Carlsbad, CA, USA) in OPTI-MEM I reduced-serum medium (Gibco), according to the manufacturer’s protocol. For loss-of-function experiments, NF1 specific siRNA (AM16708; Thermo Fisher Scientific), SRED2 specific siRNA (M-018590-00-0005; Dharmacon, Lafayette, CO, USA), and control siRNA (D-001810-01-05; Dharmacon) were used. For gain-of-function experiments, an NF1 expression plasmid (kindly prepared by Prof. Sakaguchi), a SPRED2 expression plasmid (NM_181784; Origene Technologies, Rockville, MD, USA), and a control plasmid (TP700079; Origene Technologies) were used. The cells were subsequently cultured for 48 h, and the efficacy of each transfection was validated by real-time quantitative PCR (RT-qPCR) or Western blotting as described below (Section 4.7 and Section 4.8).

### 4.4. Cell Proliferation Assay

Cells were seeded at a density of 2 × 10^3^ cells per well in a 96-well plate containing 100 µL of cell suspension. Cell proliferation was assessed using an MTT assay (Roche, Mannheim, Germany), according to the manufacturer’s instructions. The optical density (OD) at 570 nm was measured with a microplate reader. Higher absorbance values indicated greater cell proliferation. The experiments were performed in triplicate.

### 4.5. Transwell Migration and Invasion Assay

Cell migration and invasion were evaluated using uncoated Transwell membrane filters for migration assays and Matrigel-coated filters for invasion assays. The filters (pore size, 8 μm) were placed in a 24-well plate (Corning, Lowell, MA, USA). HCC1937 cells (5 × 10^5^ cells) were seeded into the upper chamber and incubated for 24 h at 37 °C. Cells that migrated or invaded to the lower surface of the membrane were fixed with methanol and stained with crystal violet. Three random low-power fields (×20 magnification) were selected per membrane to count the number of migrated cells. All experiments were performed in triplicate.

### 4.6. Scratch Assay

We performed a scratch wound healing assay to evaluate the migratory ability of MCF7 cells, as significant migration or invasion was not observed in the Transwell migration and invasion assays. MCF7 cells (2 × 10^5^) were seeded in 6-well plates and grown to confluence. The cell monolayer was scratched with a sterile 200 µL tip and incubated for 48 h. Images were captured at different time points using an inverted microscope (Olympus CKX41; Olympus, Tokyo, Japan), and wound closure was quantitated using ImageJ software. The percentage of wound closure was calculated as: (original distance–final distance)/original distance × 100. All experiments were performed in triplicate.

### 4.7. RT-qPCR

Total RNA was extracted from cultured cells using the High Pure RNA Isolation Kit (Roche, Mannheim, Germany). First-strand cDNA was synthesized from 2 μg of total RNA using the High-Capacity cDNA Reverse Transcription Kit (Thermo Fisher Scientific). RT-qPCR was performed on a StepOnePlus system (Thermo Fisher Scientific) using Taqman gene expression assay kits (SPRED2; Hs00986220_m1, GAPDH; Hs02758991_g1., Thermo Fisher Scientific). Gene expression levels were normalized to GAPDH expression.

### 4.8. Western Blotting

For total cell extraction, cells were lysed in lysis buffer supplemented with a complete protease inhibitor cocktail (Roche, Mannheim, Germany) and the Halt™ phosphatase inhibitor cocktail (Thermo Fisher Scientific). Cell lysates were incubated on ice for 30 min and centrifuged at 15,000× *g* for 10 min. Nuclear and cytoplasmic extracts were prepared using the NE-PER Nuclear and Cytoplasmic Extraction Kits (Thermo Fisher Scientific), and membrane fractions were isolated with the Mem-PER Plus Membrane Protein Extraction Kit (Thermo Fisher Scientific), according to the manufacturers’ instructions. Protein concentrations were determined using BCA Protein Assay Kit (Takara Bio, Shiga, Japan). Protein samples (15–30 μg) were separated by sodium dodecyl sulfate polyacrylamide gel electrophoresis (SDS-PAGE) (Thermo Fisher Scientific) and transferred onto polyvinylidene fluoride (PVDF) membranes. After blocking, the membranes were incubated overnight with primary antibodies, followed by incubation with horseradish peroxidase (HRP)-conjugated secondary antibodies. Target proteins were visualized using ImmunoStar LD (Wako, Osaka, Japan), and the membranes were scanned using a C-DiGit Blot Scanner (LI-COR Biotechnology, Lincoln, NE, USA). Blot images were semi-quantitated using Image Studio software (v1.54f). The antibodies used for Western blotting are listed in Table 4.

### 4.9. Immunocytochemistry (ICC) and Immunofluorescence (IF)

For ER, PR, HER2 staining, cells (1 × 10^3^) were seeded onto Lab-Tek II chamber slides (Thermo Fisher Scientific), incubated at 37 °C for 24 h, and then fixed with 10% formalin. After blocking with Dako Protein Block Serum-Free (Dako, Carpinteria, CA, USA), the samples were incubated with primary antibodies for 1 h, followed by secondary antibodies for another hour. Visualization was performed using the DAB detection system, and counterstaining was carried out with hematoxylin. For SPRED2 immunofluorescence staining, cells were seeded at a density of 1 × 10^3^ cells on Lab-Tek II chamber slides. To enhance membrane permeability, the cells were chemically skinned as previously described with slight modifications [39,40]. Briefly, the cells were rinsed with paracytosol buffer (137 mM potassium glutamate, 4 mM MgSO_4_, 3 mM ATP, 0.5 mM EGTA, 2 mM DTT, and Hepes-NaOH at pH 6.8), permeabilized with the same buffer containing 40 μg/mL of digitonin and a protease inhibitor cocktail for 15 min at 37 °C, washed with PBS, and then fixed with 4% paraformaldehyde. The fixed cells were blocked with PBS containing 1% Tween-20 and 0.1% BSA for 1 h at room temperature, followed by overnight incubation at 4 °C with an anti-SPRED2 polyclonal antibody. After washing, the cells were incubated with an Alexa Fluor 488-conjugated secondary antibody. Nuclei were counterstained using ProLong™ Gold Antifade Mountant with DAPI (Thermo Fisher Scientific). Images were acquired using a LSM780 confocal laser scanning microscope (Zeiss Microscopy, Jena, Germany).

### 4.10. Immunohistochemistry (IHC)

Paraffin-embedded tissue sections (4 μm thick) were deparaffinized, rehydrated, and treated with 3% H_2_O_2_ in methanol for SPRED2 staining or in distilled water for NF staining for 10 min at room temperature. Antigen retrieval was performed in a microwave oven using 0.1M citric acid buffer for SPRRED2 or EDTA for NF. Sections were blocked with Dako Protein Block Serum-Free (Dako, Carpinteria, CA, USA) for 1 h at room temperature. Subsequently, the sections were incubated overnight incubation at 4 °C with either an anti-human SPRED2 polyclonal antibody (Proteintech, Rosemont, IL, USA) or an anti-NF antibody (Cell Signaling Technology). After washing, the sections were incubated with Polink-2 plus HRP rabbit antibody specific enhancer, followed by polymer-HRP for rabbit IgG, and visualized using the DAB detection system. Nuclear counterstaining was performed with hematoxylin.

### 4.11. Co-Immunoprecipitation Assay (Co-IP)

Cells were lysed in Pierce^TM^ IP lysis buffer (Thermo Fisher Scientific) supplemented with Halt™ phosphatase inhibitor cocktail, incubated on ice for 30 min, and centrifuged at 15,000× *g* for 15 min. To reduce nonspecific binding, Dynabeads^TM^ protein G (Thermo Fisher Scientific) were added to the lysates and rotated for 1 h at 4 °C. After centrifugation, the lysates were incubated with the primary antibody and rotated for 2 h at 4 °C. Dynabeads^TM^ protein G were then added, and the mixture was rotated overnight at 4 °C. The beads were washed four times with lysis buffer, resuspended in 20 μL of SDS sample buffer, and heated at 100 °C for 10 min. The resulting samples were subjected to SDS–PAGE, transferred to membranes, and probed with the indicated antibodies. The antibodies used for Co-IP and Western blotting are listed in Table 4.

### 4.12. Clinical Samples

An a priori power analysis was performed to determine the required sample size for a chi-square test comparing TNBC and luminal A BC tissues. Assuming a medium effect size (Cohen’s *w* = 0.3), a two-sided α of 0.05, and a power of 0.80, the estimated total sample size was approximately 87, assuming equal allocation of approximately 44 cases per group. To account for potential unusable samples, the planned sample size was conservatively increased. A total of 94 invasive BC specimens (TNBC, *n* = 49; luminal A, *n* = 45) from patients who had not received preoperative chemotherapy between 2008 and 2012 were retrieved from the Pathology database of Okayama University Hospital (Table 5). Pathological data, including ER, PR, HER2, and Ki 67 status, were obtained from routine reports. Hematoxylin and eosin (HE)-stained sections were reviewed independently by two blinded pathologists, and corresponding paraffin blocks were used for immunohistochemistry. The study was conducted in accordance with the Declaration of Helsinki and approved by the institutional review boards of Okayama University (approved number 1710-042 and approved date 28 March 2025). Although written informed consent was not obtained, the study protocol was publicly disclosed on our website to allow patients or their families to opt out; only cases without refusal were included. The study was approved by the Institutional Review Board of Okayama University (approval number 1710-042).

### 4.13. Database Analyses

*NF1* and *SPRED2* mRNA expression in normal breast tissues, BC tissues, and metastatic tissues were obtained from TNMplot (https://tnmplot.com/analysis/ (accessed on 1 September 2025)). The prognostic significance of *NF1* and *SPRED2* mRNA expression in patients with invasive luminal A BC and TNBC were assessed using the data from the Kaplan–Meier Plotter (https://kmplot.com/analysis/ (accessed on 16 September 2025)).

### 4.14. Statistical Analysis

Statistical analyses were performed using GraphPad Prism 6.0 software (San Diego, CA, USA). The Shapiro–Wilk test was used to assess the normality of data distribution within each group, and all data met the assumptions of normality. Differences between two groups were analyzed using the two-tailed unpaired Student’s *t*-test. For immunohistochemistry data, staining categories were compared between groups using the chi-square test. Data were presented as the mean ± SEM, and a *p*-value < 0.05 was considered statistically significant.

## Figures and Tables

**Table 1:** NF staining in breast cancer patients.

	Total Cases	Negative	Low	High	*p*
TNBC	49	21 (42.9%)	20 (40.8%)	8 (16.3%)	0.0213
Luminal A	45	10 (22.2%)	21 (46.7%)	14 (31.1%)	

*p*: chi-squared test.

**Table 2:** SPRED2 staining in breast cancer patients.

	Total Cases	C−M−	C+M−	C+M+	*p*
TNBC	49	11 (22.4%)	38 (77.6%)	0 (0%)	<0.0001
Luminal A	45	6 (13.3%)	21 (46.7%)	18 (40.0%)	

C: cytoplasm, M: membrane; *p*: chi-squared test.

**Table 3:** SPRED2 staining in NF-negative and NF-positive breast cancer patients.

	NF-Negative		NF-Positive		
	C+M−	C+M+	C+M−	C+M+	*p*
TNBC	21	0	28	0	0.0213
Luminal A	10	0	17	18	

C: cytoplasm, M: membrane; *p*: chi-squared test.

**Table 4:** Primary antibodies used in this study.

Antigen	Company (Cat. Number)
*Neurofibromin* (NF)	Proteintech (27249-1-AP) ^1,2^
	Santa Cruz Biotechnology (sc-376886) ^1,2^
SPRED2	Proteintech (24091-1-AP) ^1,2^
	Santa Cruz Biotechnology (sc-517018) ^1,2^
p44/42 MAPK (ERK1/2)	Cell Signaling Technology (4695) ^1^
Phospho-p44/42 MAPK (pERK1/2)	Cell Signaling Technology (4370) ^1^
c-RAF (D4B3J)	Cell Signaling Technology (53745) ^1^
Phospho-c-RAF (Ser338) (56A6)	Cell Signaling Technology (9427) ^1^
CyclinD1	Cell Signaling Technology (92G2) ^1^
GAPDH	Cell Signaling Technology (5174) ^1^
Estrogen Receptor a (D6R2W)	Cell Signaling Technology (13258) ^1^
Progesterone Receptor A/B (D8Q2J)	Cell Signaling Technology (8757) ^1^
HER2/ErbB2 (29D8)	Cell Signaling Technology (2165) ^1^
Mouse IgG Isotype control	Cell Signaling Technology (14269S) ^1^
Normal Rabbit IgG	Cell Signaling Technology (5415S) ^1^
HRP-goat anti-rabbit IgG	Cell Signaling Technology (7074) ^1,2^
HRP-anti-mouse IgG	Cell Signaling Technology (7076) ^1,2^
Goat anti-Rabbit IgG, Alexa Fluor 488 conjugated	Thermo Fisher Scientific (A-11008) ^1^

^1^ Antibodies used for Western blotting; ^2^ Antibodies used for Co-immunoprecipitation assay. Cell Signaling Technology: Danvers, MA, USA. Proteintech: Rosemont, IL, USA. Thermo Fisher Scientific: Waltham, MA, USA. Santa Cruz Biotechnology: Dallas, TX, USA.

**Table 5:** The clinicopathological characteristics of the patients with invasive breast cancer.

Characteristics		n	(%)
Age	≤49	29	30.85
	≥50	65	69.15
Tumor size	pT1 (≤5 mm)	6	6.38
	pT1b (6–10 mm)	14	14.89
	pT1c (10–20 mm)	46	48.94
	pT2 (20–50 mm)	24	25.53
	pT3 (>50 mm)	4	4.25
Lymph node metastasis	pN0	69	73.40
	pN1	15	15.96
	pN2	3	3.19
	pN3	5	5.32
	not accessible	2	2.13
Histologic grade	Grade 1 and 2	52	55.32
	Grade 3	42	44.68
Estrogen receptor	Negative	49	52.13
	Positive	45	47.87
Progesterone receptor	Negative	49	52.13
	Positive	45	47.87
Her2 overexpression	Negative	94	100
	Positive	0	0
Ki67 index	≤20%	51	54.26
	>20%	43	45.74
Intrinsic subtypes	Luminal A	45	47.87
	Triple negative	49	52.13
Histologic type	No special type (NST)	94	100
	Invasive lobular carcinoma	0	0
	Mucinous carcinoma	0	0
	Invasive micropapillary carcinoma	0	0
	Other special types	0	0
Outcome	Disease free survival ^1^	70	74.47
	Overall survival ^2^	74	78.72
	Death	19	15.79

^1^ Median follow up for disease free survival was 2607 days. ^2^ Median follow up for overall survival was 3681 days.

## Data Availability

The data that support the findings of this study are available from the corresponding author upon reasonable request.

## Supplementary Materials

The following supporting information can be downloaded at: https://www.mdpi.com/article/10.3390/ijms262010072/s1.

## Abbreviations

The following abbreviations are used in this manuscript:

NF1	neurofibromatosis type 1
NF	Neurofibromin
SPRED	Sprouty-related EVH1 domain-containing protein
BC	breast cancer
IHC	immunohistochemistry
TNBC	triple-negative breast cancer
ER	estrogen receptor
PR	progesterone receptor
HER2	human epidermal growth factor receptor 2
HCC	hepatocellular carcinoma
siRNA	small interfering RNA
KD	knockdown
OE	overexpression
