# Supplementary material for: Neurofibromin Encoded by the Neurofibromatosis Type 1 (NF1) Gene Promotes the Membrane Translocation of SPRED2, Thereby Inhibiting the ERK Pathway in Breast Cancer Cells
Source: Int J Mol Sci. 2025 Oct 16;26(20):10072. doi: 10.3390/ijms262010072 (PMC12563662; doi:10.3390/ijms262010072)
Supplement: Supplementary file 1 [file ijms-26-10072-s001.zip › ijms-3918548-supplementary.pdf]

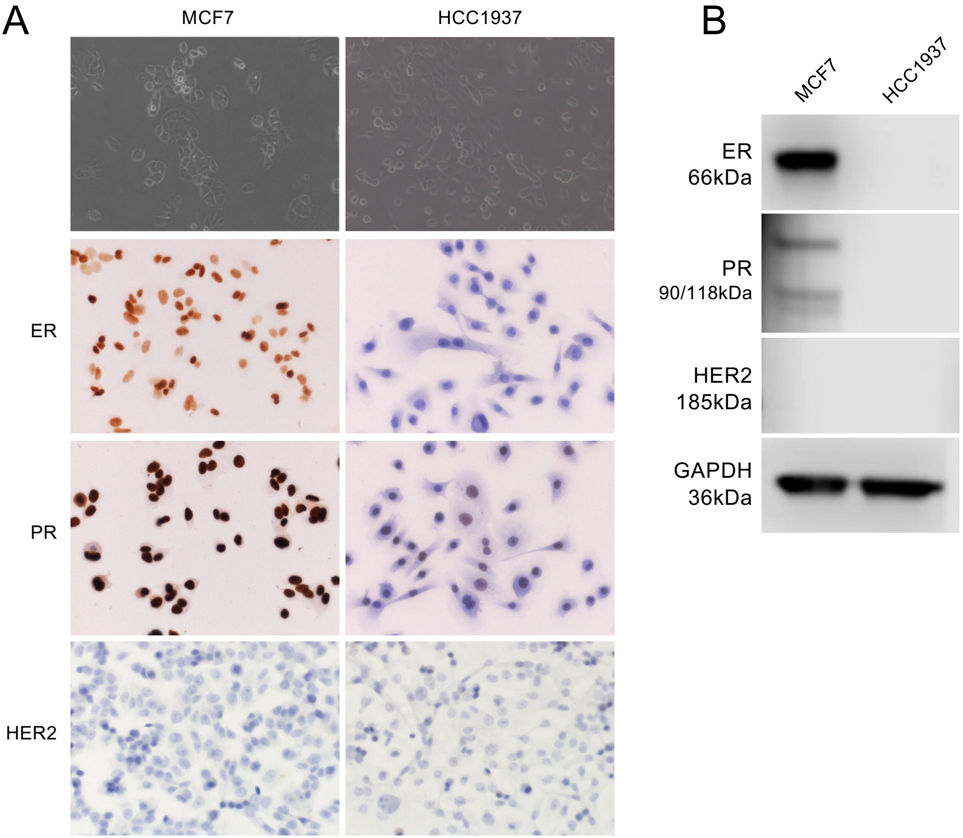

Figure S1. The molecular subtypes of MCF7 and HCC1937 cells. (A) MCF7 and HCC1937 cells cultured on Lab-Tek II Slide (8 Chamber, Electron Microscopy Sciences, Hatfield, PA, USA) were fixed in 95% ethanol and immunostained with antibodies against estrogen receptor (ER), progesterone receptor (PR) or human epidermal growth factor receptor 2 (HER2). Representative photos are shown. Positive cells were shown in brown. (B) The levels of each protein in MCF7 and HCC1937 cells were assessed by Western blotting. Representative images are shown.

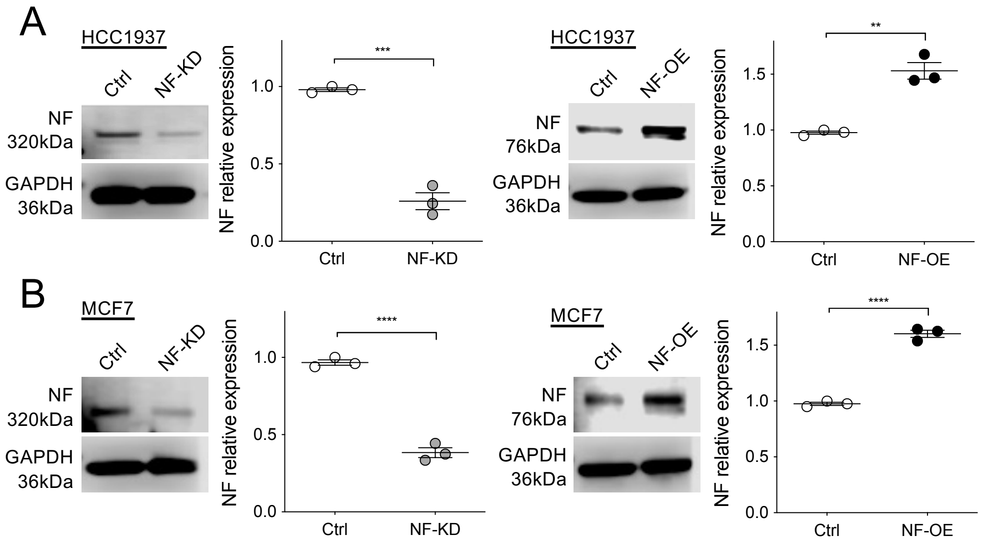

Figure S2. NF expression after NF depletion or overexpression. NF was knocked down (NF-KD) or overexpressed (NF-OE) in HCC1937 (A) and MCF7 (B) cells. Cell lysates were prepared, and the presence of each protein was evaluated by Western blotting. Band densities were digitized and semi-quantitated (3 independent experiments, each). $^{**}p < 0.01$, $^{***}p < 0.001$, $^{****}p < 0.0001$, two-tailed unpaired t-test.

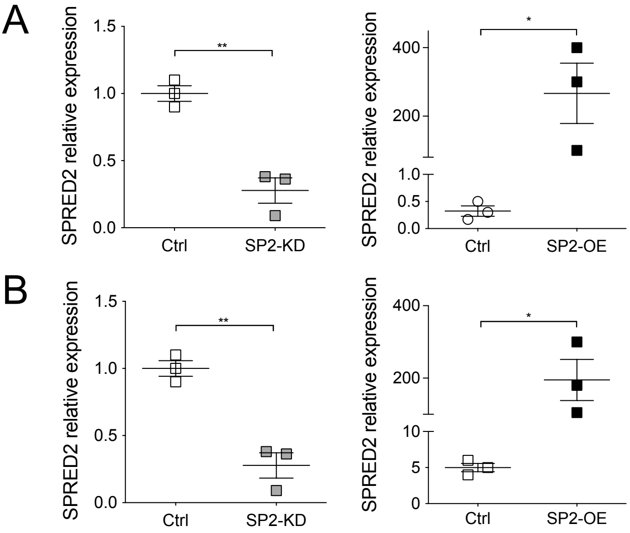

Figure S3. *SPRED2* mRNA expression after *SPRED2* depletion or overexpression. SPRED2 (SP2) was knocked down (SP2-KD) or overexpressed (SP2-OE) in HCC1937 (A) and MCF7 (B) cells. *SPRED2* mRNA expressions in the cells were examined by RT-qPCR (3 independent experiments, each). $^{*}p < 0.05$, $^{**}p < 0.01$, two-tailed unpaired t-test.

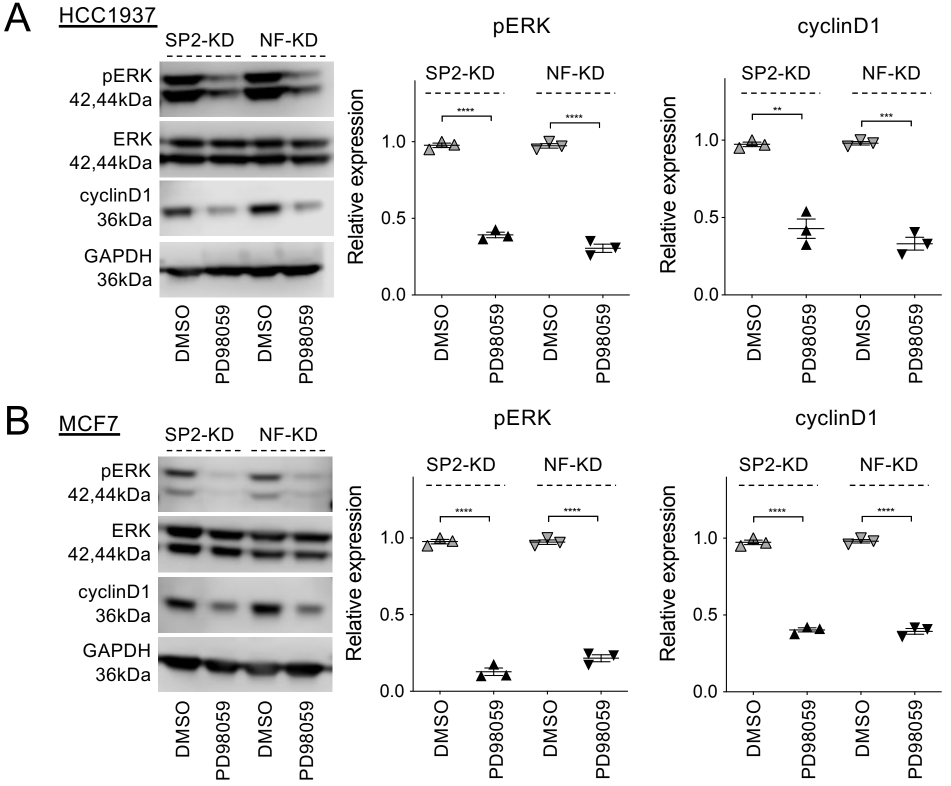

Figure S4. Increased ERK activation and cyclin D1 levels following SPRED2 or NF deletion were reduced by PD98059. SPRED2 (SP2) or NF was knocked down (SP2-KD/ NF-KD) in HCC1937 (A) and MCF7 (B) cells. Cells were then treated with the MEK/ERK inhibitor PD98059 (20 μM; Thermo Fisher Scientific, MA, USA) for 24 hours. DMSO was used as a control. Cell lysates were prepared, and the presence of each protein was evaluated by Western blotting. Band densities were digitized and semi-quantitated (3 independent experiments, each). $^{**}p < 0.01$, $^{***}p < 0.001$, $^{****}p < 0.0001$, two-tailed unpaired t-test.
